# Effect of pulse targeting strategy on Er:YAG laser ablation rate and efficiency in ex vivo cancellous bone

**DOI:** 10.1007/s10103-026-05075-0

**Published:** 2026-10-24

**Authors:** Sam Withers, Joshua Troy, Brett Robertson, Riaz Khan, Nathan Pavlos

**Affiliations:** 1 School of Biomedical Sciences, The University of Western Australia, Perth, Australia; 2 ArthroLase, Perth, Australia; 3 School of Physics, Mathematics and Computing, The University of Western Australia, Perth, Australia

**Keywords:** Laser-tissue interaction, Laser osteotomy, Hard tissue ablation, Thermomechanical ablation, Scanning strategy, Laser arthroplasty

## Abstract

**Purpose:**

Lasers are an attractive alternative to mechanical tools for bone preparation, but clinical adoption is limited by low removal rates. This study investigated whether spatiotemporal sequencing of Er:YAG pulses can improve thermomechanical bone ablation, and how pulse separation and delivery order affect ablation rate, efficiency, and heating.

**Methods:**

A prototype Er:YAG system, with galvanometer scanning, ablated cancellous bone in eight ex vivo lamb femurs. Arm 1 varied the separation between consecutive pulses (0.175 to 0.275 mm). Arm 2 compared four targeting patterns within a fixed pulse grid, maintaining constant fluence and total energy for three of the four patterns. Thermal imaging and pre/post confocal scans quantified heating and volumetric removal.

**Results:**

Ablation rate was consistent across pulse separations (521 to 552mm^3^/min), while layer depth scaled linearly with deposited energy (R^2^ = 0.94), decoupling depth control from throughput. Targeting strategy significantly affected ablation efficiency, ranging from 0.19–0.32mm^3^/J (*p* < 0.001). Hatching was fastest and most efficient, sustaining consistently high peak surface temperatures (200–250 °C) but lower mean surface temperatures than either scatter strategy.

**Conclusion:**

Ablation efficiency varied with pulse order under controlled conditions, indicating a dependence beyond single pulse dynamics. We propose inter-pulse thermal coupling, whereby residual heat from a preceding pulse lowers the energy required by the next. Optimising the targeting strategy achieved the fastest reported removal rate for cancellous bone, identifying pulse sequencing as a lever for more efficient ablation.

**Supplementary Information:**

The online version contains supplementary material available at https://doi.org/10.1007/s10103-026-05075-0.

## Introduction

Mechanical tools, including oscillating saws, burs, and reamers, remain the gold standard for bulk bone removal in arthroplasty and trauma procedures within orthopaedic surgery. While these instruments have evolved over centuries and are increasingly integrated with robotic and computer navigation systems [1, 2], fundamental limitations persist. Even with robotic assistance, the inherent constraints of saw-based techniques can result in cutting inaccuracies that compromise procedural precision [3]. Furthermore, these tools generate significant frictional heat during operation, which can lead to osteonecrosis in the surrounding tissue [4, 5].

Laser systems present a promising alternative bone removal modality, with demonstrated clinical success across various medical fields including ophthalmology and dentistry [6]. The dual requirements of large volume removal combined with the inherent mechanical strength of bone have made it challenging to achieve clinically relevant removal rates. For bone ablation, the laser wavelength must be selected to maximise optical absorption which enables efficient conversion of optical energy to thermal and mechanical energy within a shallow surface layer, driving thermomechanical ablation. The Er:YAG laser emitting at 2.94 μm is particularly well-suited for hard tissue applications due to strong absorption by water and hydroxyapatite, the primary constituents of bone, with an optical penetration depth of 2 μm [7].

To date, mid-infrared laser systems have most commonly been deployed for cutting and drilling applications, mimicking the linear removal approaches of traditional mechanical tools [8–12]. While many studies have demonstrated improvements in healing response relative to existing tools [7], the critical limitation of removal speed remains unresolved. A peak ablation rate of 384 mm^3^/min [13], reported as the current ceiling of the field [14], would require substantial improvement to achieve commercial viability for larger resection procedures [15].

Laser beam scanning presents unique opportunities for true three-dimensional bone removal, moving beyond conventional planar cutting paradigms. Lee et al. outlined the potential for lasers to perform CNC-like cutting of tissue in accordance with a surgical plan [16]. This approach involves delivering discrete laser pulses at specified spatial coordinates, systematically removing material in accordance with pre-planned three-dimensional geometries. Early outcomes have been demonstrated by Werner and colleagues using scanning CO_2_ lasers for freeform volumetric removal of bone [17–19]. Ultrafast near-infrared (NIR) lasers have also garnered interest for their potential utility in bone shaping [20–22]. Yin et al. a decade ago demonstrated the potential for integrating sensor feedback to selectively shape bone [22]. While these studies established proof-of-concept for laser scanning approaches, they deployed CO_2_ and NIR lasers that exhibited low removal rates and potential for compromising tissue health [7].

The use of laser beam scanning provides not only macro control of how laser pulses can be organised, but also the sequence in which pulses across a layer are delivered. In manufacturing, spatial targeting strategies play a critical role in optimising material removal rates and mitigating heat accumulation [23–27]. In subtractive manufacturing processes, scan strategies have been demonstrated to greatly impact removal rates [27], with double the removal rate observed through targeting optimisations alone [28]. Additive manufacturing, in particular selective laser melting (SLM), has observed increased strength of manufactured parts through the use of more complex scanning strategies through better thermal control of the production process [25].

The industrial applications typically operate ultrafast lasers, meanwhile mid-infrared lasers such as the Er:YAG laser, well suited to soft and hard tissue ablation, are typically constrained to the microsecond domain [13, 29]. In the microsecond pulse range, the mechanism of ablation is different from ultrafast lasers with pulses in the pico or femtosecond range. Longer pulses mean medical applications are often driven by thermomechanical ablation of bone in which thermal confinement is not achieved [30]. As such, the sequence and spatial separation of laser pulses can impact how thermal interactions occur. To our knowledge, no systematic research has investigated optimal targeting patterns or pulse separation strategies specifically designed to maximise thermomechanical ablation performance in biological tissue removal applications.

Here, using a prototype Er:YAG laser arthroplasty system, we systematically investigated optimal approaches for spatiotemporal laser pulse targeting to achieve efficient and thermally regulated laser bone ablation.

## Materials and methods

### Prototype system

**Fig. 1:**
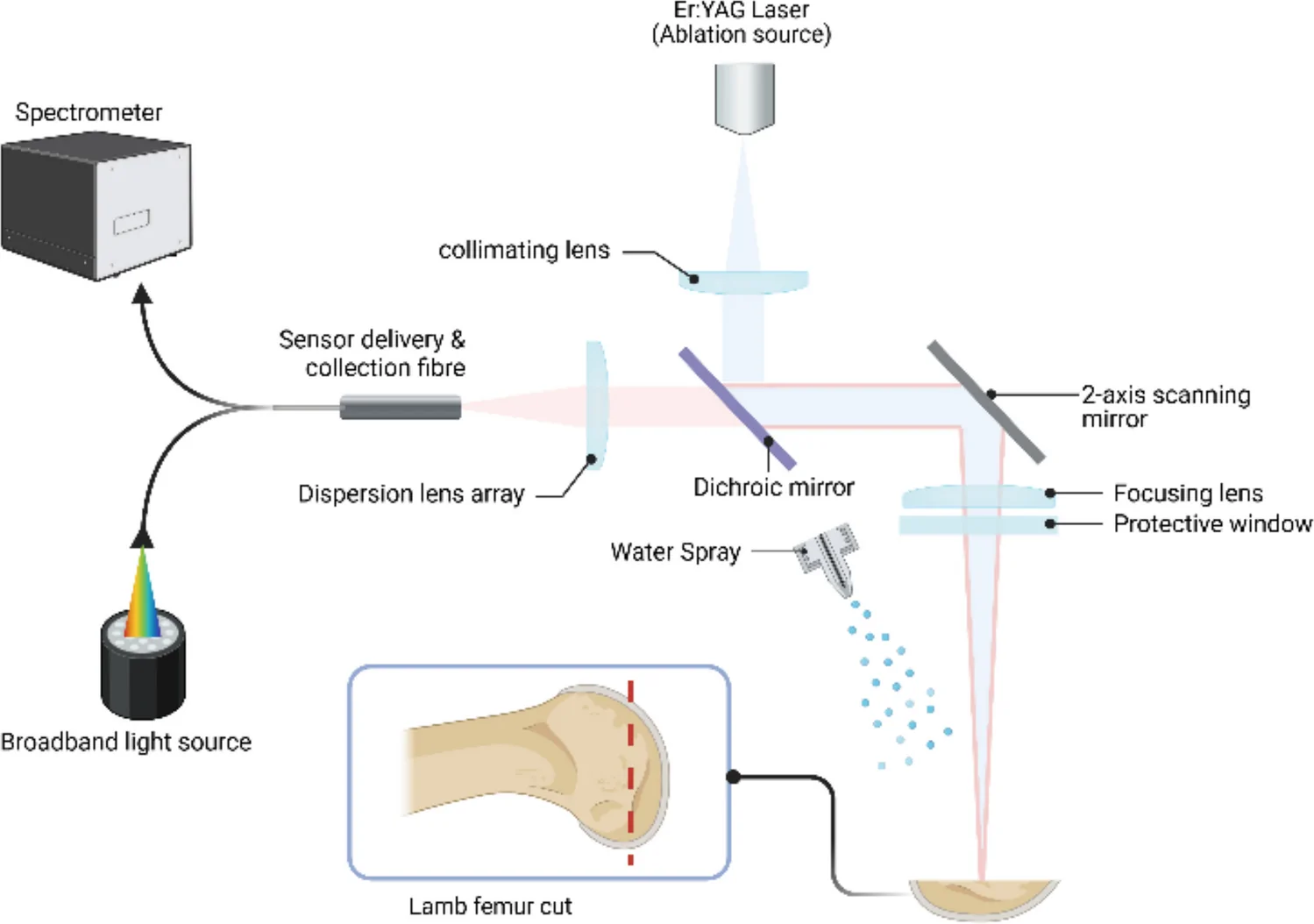
Experimental schema of the prototype laser arthroplasty system

Ablation experiments were performed using a prototype laser arthroplasty system that integrates an Er:YAG laser (Pantec Biosolutions) emitting at 2.94 μm with a custom chromatic confocal sensor and targeting optics (Fig. 1). The Er:YAG beam was collimated to a 31 mm diameter by a CaF₂ plano-convex collimating lens, reflected off a dichroic mirror, steered by a two-axis galvanometer scanner (protected-silver mirrors, Thorlabs), and focused by an AR-coated ZnSe plano-convex lens to a 150 mm working distance. Laser firing was coordinated with galvanometer motion, so pulses were delivered to discrete coordinates defined by the control software.The prototype system delivered an average power of 29 W to the bone surface at a 350 Hz pulse repetition rate and 200 μs pulse duration. Beam quality was characterized across a range of duty cycles on a separate benchtop setup using the knife-edge method (ISO 11146–1:2021). Interpolating from these measurements gave an M2 of 27.6 at the nominal operating parameters. The spot size (1/e^2^) was calculated using the formula ${d}_{o}=\frac{4{M}^{2}\lambda f}{\pi D}$, resulting in a spot size of 500µm producing a fluence of 42.3 J/cm^2^ at the 150 mm focal plane. The depth of field, defined as twice the Rayleigh range, was calculated as 4.8 mm using $DOF=\frac{{\pi d}_{0}^{2}}{{2M}^{2}\lambda }$. A water-spray system adjacent to the optical output used two nozzles to deliver saline to the ablation site.

A custom chromatic confocal sensor provided non-contact surface topography measurement within the optical system. Near-infrared light from a custom supercontinuum source, delivered through a double-clad fibre (Castor Optics) and dispersed axially by the chromatic lens system, gave a 15 mm axial measurement range. A spectrometer (Avantes; 680–980 nm, 0.93 nm FWHM) determined surface position from the peak reflected wavelength, giving a spatial resolution of 10 μm. Surface maps were acquired by triggering the spectrometer at discrete cartesian coordinates as the galvanometer mirrors traced a raster pattern across the surface.

The sensor shared the ablation beam path via a dichroic beamsplitter that transmitted the measurement beam while reflecting the 2.94 μm ablation wavelength. This coaxial, focal plane centred configuration ensured depth measurements were acquired from the exact ablation site, free of parallax errors.

The optical assembly was mounted on a six-axis robotic arm, its spatial position tracked by a marker-based optical navigation camera: a marker constellation fixed to the assembly defined the end-effector pose, while the sample location was registered with a pointer tool fitted with optical markers, together positioning the robot so the focal plane coincided with the sample surface.

### Sample preparation

**Fig. 2:**
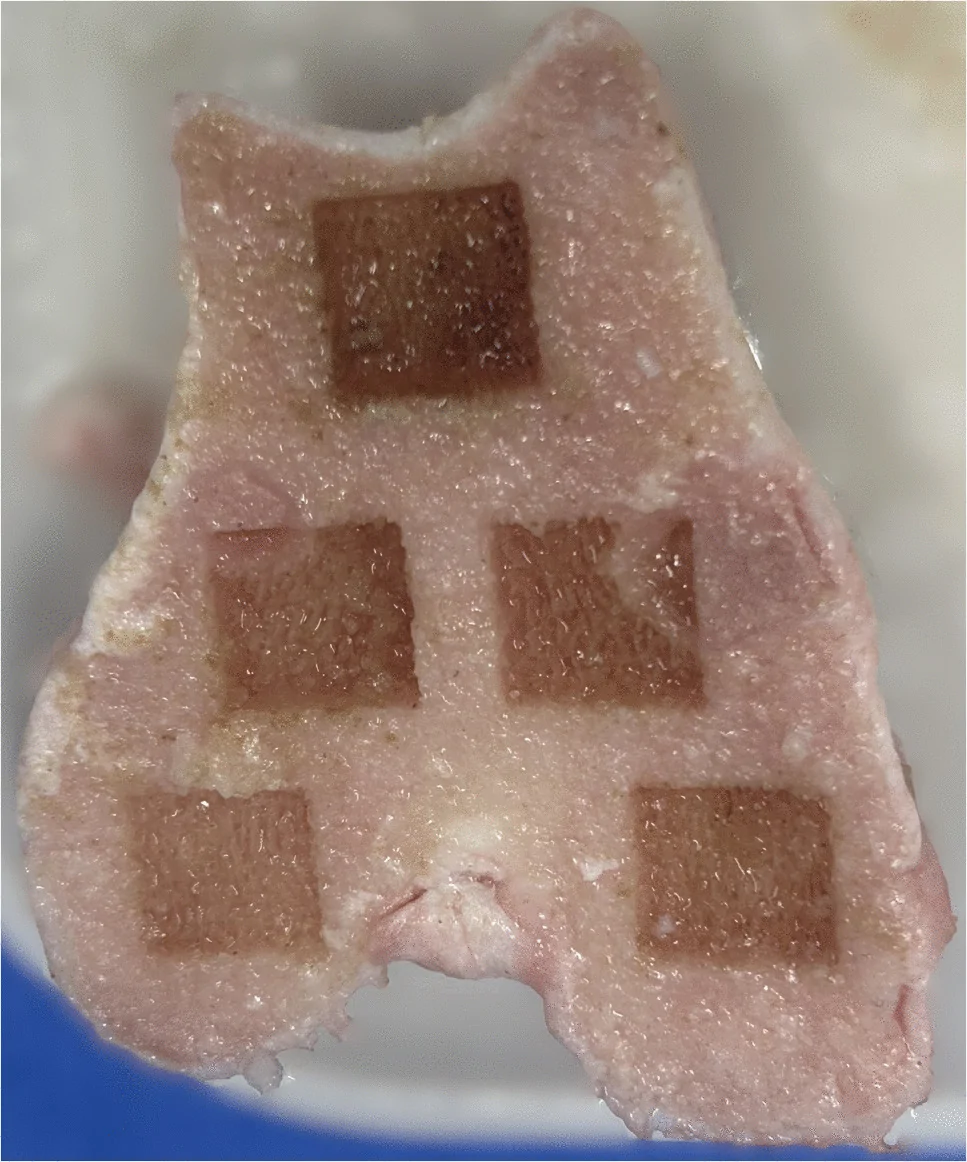
Representative lamb sample following arm 1 bone ablations

Eight lamb femur specimens were sourced and subsequently frozen at −20 °C prior to experimentation. Four specimens were allocated to each experimental arm. The distal femur was cut to expose a flat cancellous bone surface, making efforts to avoid the growth plate due to its unrepresentatively dense structure. Ovine bone is a well-established surrogate for musculoskeletal research with comparable cancellous bone structure and mineral composition [31]. We tested cancellous bone as it is the predominant bone type in the epiphyses resected during arthroplasty [32]. Prior to experimentation, each sample was thawed for one hour in ambient conditions. We configured each specimen with five 10 mm × 10 mm ablation sites and randomised experimental conditions across sites, allocating one site to each of the five pulse separations in Arm 1 (see Fig. 2) and one to each of the four targeting strategies in Arm 2 (*n* = 4). For arm 2, one site went unused.

### Ablation protocol

For each ablation site, three layers were ablated with 0.75 mm focal plane adjustments each layer to ensure all ablation layers remained within the laser's depth of field. Samples were irrigated via two spray nozzles affixed adjacent to the output window. A 1.1 s spray pulse delivered 1 ml of saline to the ablation site immediately before each layer. The spray was not active during laser exposure, as surface water would occlude the thermal signature of the bone and prevent the intra-layer thermal dynamics from being resolved. The same spray volume and timing were applied to every layer of each site. Following completion of each site, the bone surface was dried to remove excess water and mitigate water accumulation across the specimen.

Two experimental arms were designed to investigate different aspects of laser targeting optimisation. Arm 1 examined the effect of pulse separation across ablation layers, with separation defined as distance between consecutive pulses. A hatching targeting strategy was used, consisting of a sequential line-by-line scanning. Through preliminary testing, pulse separations (d) of 0.175–0.275 mm (34%—56% area overlap) were determined most relevant.

Arm 2 compared four targeting strategies that share a fixed grid of pulse locations but differ in the order in which those locations are addressed. Pulse separation of 0.225 mm was used across this arm, with the total number of pulses held constant. These ranged from sequential line-by-line delivery to scatter approaches in which the site is divided into many sub-regions. Table 1 defines each strategy, and Visualisation 1 presents a burn-paper demonstration of each pattern.

**Table 1:** Targeting strategy definitions and characteristics

Pattern	Explanation	Time between adjacent pulses (ms)		Average Power (W)	Pulse Fluence (J/cm^2^)	Repetition Rate (Hz)
		Fast axis	Slow axis			
Hatching	Sequential line-by-line scanning with snaking pattern	2.9	125.7	29	42.3	350
Interlaced lines	Line scans broken into 5 groups which are iteratively cycled through	2.9	1131.4	29	42.3	350
Low-dispersion scatter	The ablation region is broken into a group of 5 × 5 squares, in which pulses are hatched through each group	84.7	762.7	26.3	45.5	295
High-dispersion scatter	The ablation region is broken into a group of 15 × 15 squares, in which pulses are hatched through each group	642.9	1928.6	29	42.3	350

Pulses are sequenced first along the fast axis, so the nearest pulse along a line arrives after only a short interval. The slow axis runs across successive lines, so the nearest pulse on the adjacent line arrives after a much longer interval. In the scatter strategies this ordering is applied within each sub-region rather than across the whole ablation area.

Due to the number of large galvanometer movements required, low-dispersion scatter was performed at a reduced frequency of 295 Hz and a corresponding average power of 26.3W. For the scatter strategies, the slow-axis interval equals the fast-axis interval multiplied by the number of pulses per track within each sub-region (nine for low-dispersion and three for high-dispersion), reflecting their different sub-region sizes.

### Thermal imaging

Surface thermal imaging of bone provided insight into thermomechanical processes and residual heating into bone tissue. Thermal analysis was performed using a FLIR E85 thermal camera (uncooled microbolometer, 30 frames per second) positioned approximately 30 cm from the bone surface. This frame rate provides time-averaged measurements of surface temperature; intra-pulse thermal dynamics and subsurface heating are not resolved. An emissivity value of 0.95 was used for cancellous bone, consistent with the experimentally determined range of 0.95–0.97 reported for bone tissue [33].

A 10 mm x10mm region of interest (ROI) was defined over the ablation area. Within this ROI, we computed the mean surface temperature and the peak surface temperature for each frame. The mean surface temperature provides an estimate of residual heat distributed across the surface, and therefore a proxy for heat transferred to surrounding bone tissue. Peak surface temperature was measured to capture transient heating associated with the ablation process. Peak surface temperatures are reported for the first ablation layer, which provided the most controlled conditions for measuring ablation dynamics. Mean surface temperature is reported for the final layer to assess heat accumulation over the course of ablation.

### Volumetric removal assessment

We evaluated ablation depth using the inline chromatic confocal sensor, scanning each site before and after ablation. Pre- and post-ablation surface scans were acquired across each 10 × 10 mm site using a 0.25 mm sampling grid (1600 measurement points per site). Because water pooling at the site edges made depth measurements unrepresentative, a central 6 × 6 mm measurement ROI was selected on each post-ablation surface, and ablation depth was taken as the mean nearest distance from each measurement ROI point to the pre-ablation surface.

Removal rates (mm^3^/min) were derived from the per-pulse volume (layer depth × pulse separation^2^), scaled by the number of pulses delivered per minute. Removal rates exclude spray and interlayer-movement time, using total lasing time only, for the clearest assessment of ablation parameter effects.

### Statistical analysis

Analyses were performed in Python 3.14.3 using statsmodels 0.15.0 and SciPy 1.17.1. As each specimen received every condition within its arm, specimen was included as a fixed blocking factor in all models. In Arm 1 (*n* = 20 sites), layer depth was regressed on energy density (J/mm^2^, calculated as pulse energy divided by the square of pulse separation) by ordinary least squares, with the slope (mm3/J) taken as ablation efficiency. In Arm 2 (*n* = 16 sites), efficiency was compared by two-way additive ANOVA with targeting strategy and specimen as factors, followed by Tukey's HSD. Efficiency rather than removal rate was compared to normalize for differences in delivered energy under low-dispersion scatter. Statistical significance was set at *p* < 0.05.

## Results

### Removal rate and efficiency

**Fig. 3:**
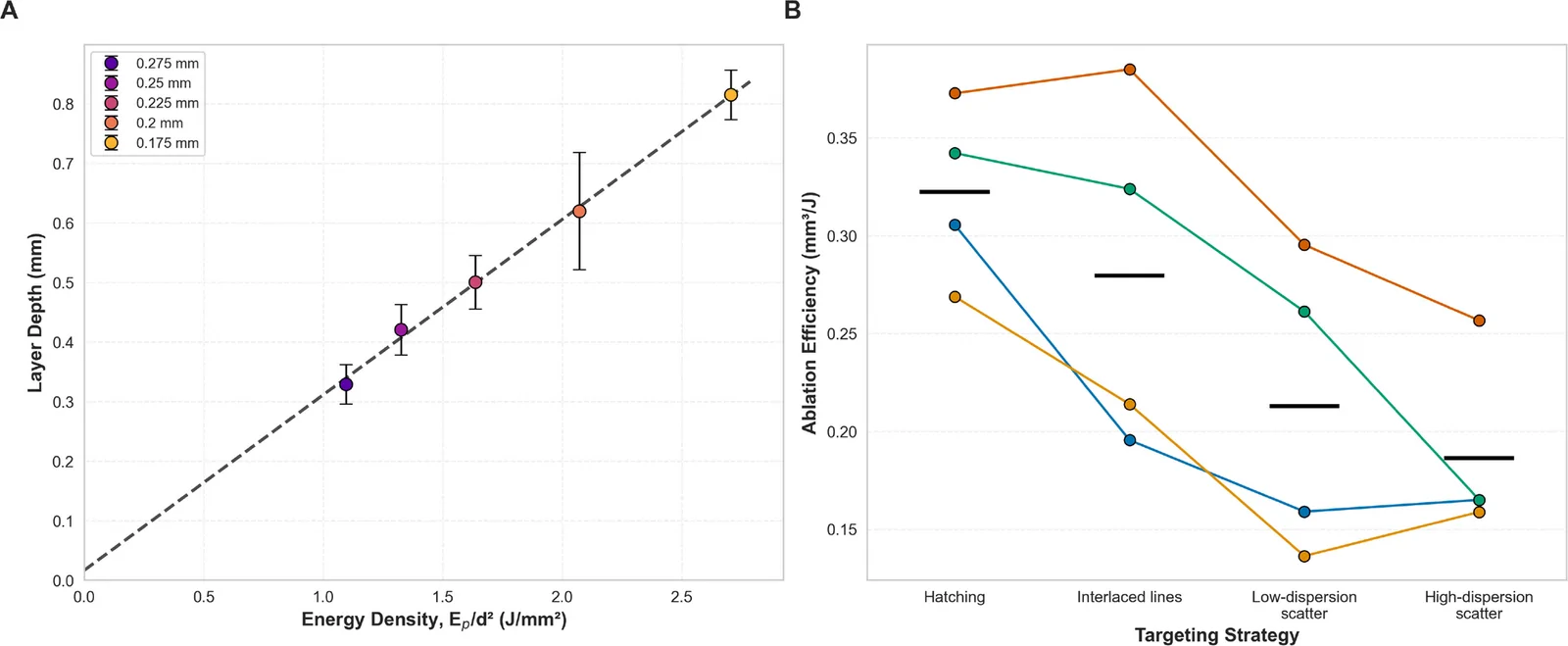
**A** Energy density (modulated by pulse separation) vs ablation layer depth with the dashed line is a ordinary least-squares regression (R2 = 0.94). **B** Ablation efficiency by targeting strategy. Coloured points joined by lines show individual specimens; horizontal bars show the group mean

Table 2 provides a summary of the measured ablation depths and derived layer depth, removal rate and ablation efficiency across both experimental arms. Tighter pulse separations observed increases in ablation depth proportional to energy density (Fig. 3a, R^2^ = 0.94, *p* < 0.001) and the regression slope gave an ablation efficiency of 0.294mm^3^/J. The intercept did not differ significantly from zero (0.017 mm, *p* = 0.66). This provides a tunable parameter for cut depths at no cost to removal rate or efficiency.

**Table 2:** Removal rates across the two experimental arms

Experimental Arm	Independent Variable	Total Ablation Depth (mm)	Removal Rate (mm^3^/min)	Layer Depth	Ablation Efficiency (mm^3^/J)
Pulse Separation (mm)	0.175	2.45 ± 0.12	524 ± 26	0.82 ± 0.04	0.30 ± 0.02
	0.2	1.86 ± 0.30	521 ± 83	0.62 ± 0.10	0.30 ± 0.05
	0.225	1.50 ± 0.13	532 ± 48	0.50 ± 0.04	0.31 ± 0.03
	0.25	1.26 ± 0.13	552 ± 56	0.42 ± 0.04	0.32 ± 0.03
	0.275	0.99 ± 0.10	523 ± 52	0.33 ± 0.03	0.30 ± 0.03
Pattern	Hatching	1.58 ± 0.22	559 ± 79	0.53 ± 0.07	0.32 ± 0.05
	Interlaced lines	1.38 ± 0.44	487 ± 157	0.46 ± 0.15	0.28 ± 0.09
	Low-dispersion scatter	1.13 ± 0.40	336 ± 121	0.38 ± 0.14	0.21 ± 0.08
	High-dispersion scatter	0.91 ± 0.23	324 ± 82	0.30 ± 0.08	0.19 ± 0.05

Figure 3B presents the ablation efficiency of each targeting strategy across specimens. Ablation efficiency differed significantly between strategies (*p* < 0.001), with the localised strategies outperforming the scatter strategies. Specimens also influenced ablation efficiency in this arm (*p* < 0.001), accounting for the wide spread of results observed in Table 2.

Tukey HSD pairwise comparisons are presented in Table 3. Hatching was more efficient than both scatter strategies, and interlaced lines more efficient than high-dispersion scatter. Restricting the comparison to the three power-matched strategies, excluding low-dispersion scatter which ran at slightly lower average power but higher fluence, gave the same conclusions (p = 0.004).

**Table 3:** Tukey HSD pairwise comparisons of ablation efficiency between targeting strategies

Comparison	Mean Diff	95% CI Lower	95% CI Upper	*p* value
Hatching and Interlaced Lines	0.041	−0.030	0.111	0.332
Hatching and Low-disp. scatter	0.108	0.038	0.179	**0.004**
Hatching and High-disp. scatter	0.135	0.065	0.206	**< 0.001**
Interlaced and Low-disp. scatter	0.068	−0.003	0.138	0.061
Interlaced and High-disp. scatter	0.095	0.024	0.165	**0.01**
Low-disp. scatter and High-disp. scatter	0.027	−0.043	0.098	0.642

Differences are first minus second strategy. *p*-values and simultaneous 95% CIs are adjusted for all six comparisons. Bold: *p* < 0.05

### Surface temperature

**Fig. 4:**
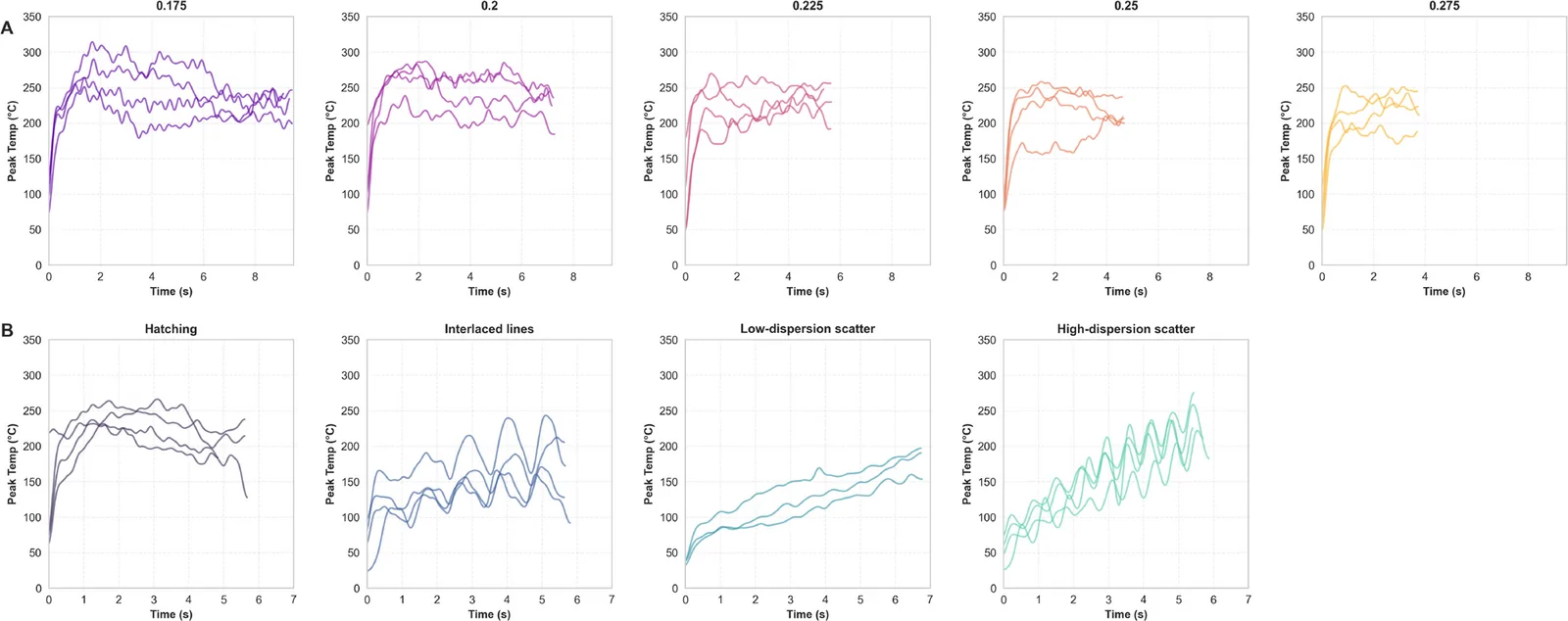
Peak surface temperature profiles during first layer ablation across each trial. (A) Peak pixel temperatures for hatching at different pulse separations (Arm 1). (B) Peak pixel temperatures for different ablation patterns (Arm 2)

Hatching and all pulse separations maintained stable, high peak surface temperatures, whereas the other three strategies ran generally lower (Fig. 4b). Interlaced lines and high-dispersion scatter showed peaks and troughs that trended upward across the layer, corresponding to their 5 and 9 passes; the many passes of low-dispersion scatter could not be resolved.

**Fig. 5:**
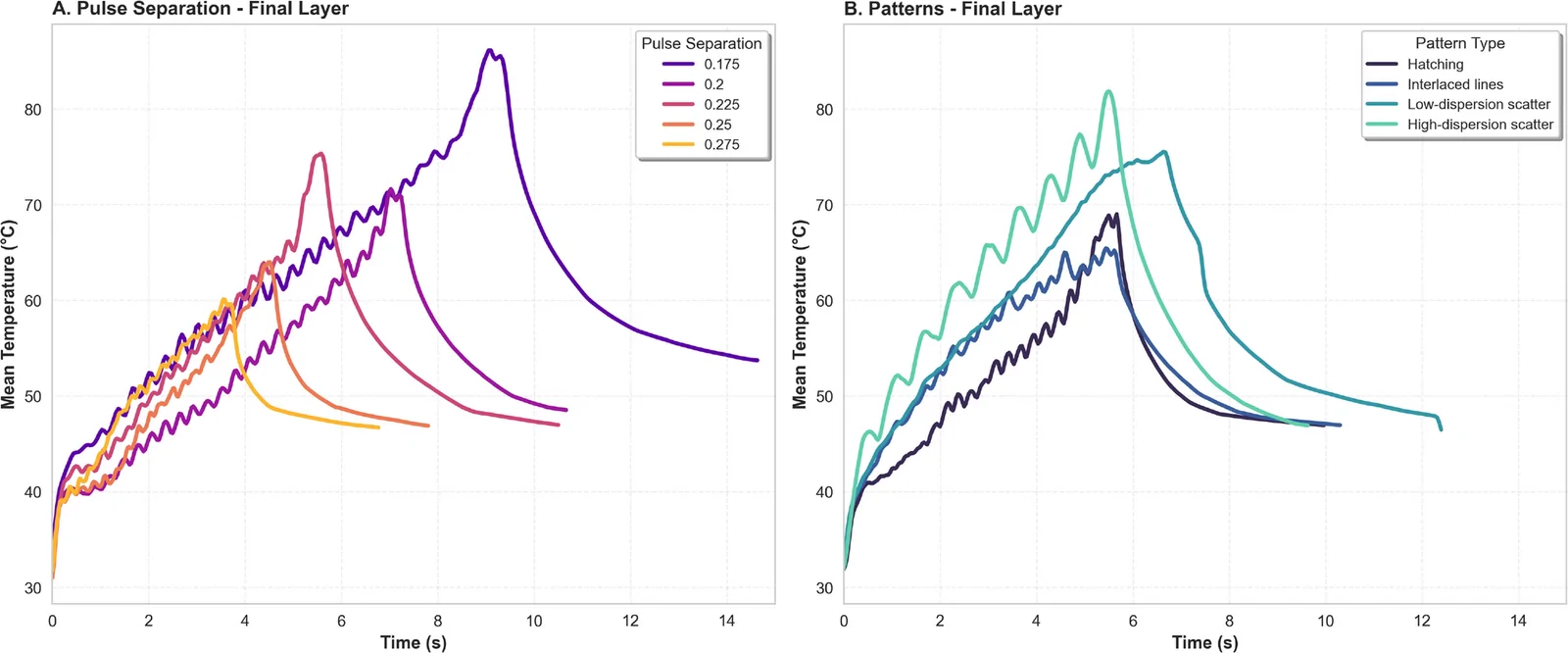
Average temperature profiles across the region of interest (ROI) during final layer ablation, with decay plotted for five seconds following completion of the layer. **A** Mean surface temperatures for different pulse separations (Arm 1). **B** Mean surface temperatures for different ablation patterns (Arm 2)

Mean ROI temperature rose throughout each layer, peaked on completion and decayed rapidly once lasing ceased (Fig. 5). In Arm 1, tighter separations deposited more energy per unit area and produced longer layers, although 0.225 mm departed from this trend (Fig. 5a). In Arm 2, high-dispersion scatter reached the highest mean temperatures, with oscillations matching successive passes, while hatching and interlaced lines remained lower (Fig. 5b). The extended duration but greater total energy deposited in low-dispersion scatter must also be noted.

## Discussion

The removal rates achieved in this study (peak 559mm^3^/min) are greater than previously reported values for laser bone ablation [14]. This improvement was enabled by high-power delivery facilitated by laser targeting, though the use of cancellous rather than denser cortical bone as the target tissue likely contributes. In this paradigm, distributed energy delivery likely sustains a more efficient thermomechanical removal process, consistent with the high ablation efficiency observed in our experiments (0.30–0.32mm^3^/J). The results further suggest that the spatiotemporal sequence of pulse delivery influences ablation efficiency, consistent with inter-pulse thermal coupling, a mechanism discussed in detail below. If so, coupling may offer further avenues for performance optimisation as laser repetition rates increase.

A notable finding from a translational standpoint is the decoupling of removal rate from layer depth control. Removal rate remained consistent across the range of pulse separations tested, while layer depth scaled linearly with deposited energy (Fig. 3a). This independence suggests that pulse density can be adjusted to modulate depth-per-layer without compromising throughput, effectively providing a tuneable precision parameter at no cost to speed. In three-dimensional bone preparation workflows, where material must be removed to variable depths across complex, nonplanar anatomies according to a pre-operative plan, this tunability is of considerable practical value. This is well suited to layer-wise bone shaping workflows, where each pass removes a prescribed depth according to the surgical plan.

By contrast, targeting strategy substantially affected efficiency. Across the three power-matched strategies (hatching, interlaced lines and high-dispersion scatter), ablation efficiency ranged from 0.19 to 0.32mm^3^/J. Because these strategies delivered the same number of pulses, at the same fluence, to the same grid of locations, this variation cannot be attributed to differences in energy deposition. Low-dispersion scatter, which ran at slightly lower average power and higher fluence, fell within this range (0.21mm^3^/J). The strategies differed principally in the spatiotemporal order in which pulses were delivered, indicating that delivery sequence is a determinant of how efficiently bone is removed.

We hypothesise that this dependence arises from inter-pulse thermal coupling. In the microsecond pulse regime, ablation proceeds thermomechanically through pressure build-up, phase explosion, and material ejection [34]. When a pulse arrives at tissue still carrying residual heat from a neighbouring pulse, its effective ablation threshold is lowered and it removes more material. Because that ejected material carries its heat away, rapid localised delivery should remove more material per pulse while leaving the tissue cooler. We use “inter-pulse thermal coupling” to denote this interaction between successive neighbouring pulses, as distinct from the “heat accumulation” that builds up over many pulses at a single site [24, 35, 36]. Coupling requires that residual heat is:

(i) a meaningful fraction of the energy needed for ablation and
(ii) still within the next pulse’s ablation volume when that pulse arrives.

On (i), the greatest observed efficiency (0.32mm^3^/J) bounds the heat of ablation, the reciprocal of efficiency, below 3.1 J/mm^3^. Peak surface temperatures during hatching typically exceeded 200 °C above baseline (Fig. 4), which for a marrow-filled trabecular density of 1.26 g/cm^3^ and specific heat of 2.35 J/g/°C [37, 38] implies a residual volumetric energy density of roughly 0.6 J/mm^3^, a fifth of the energy required for ablation. Both estimates are conservative: the 30fps camera underestimates the temperature rise from each 200 µs pulse, and conductive losses and plume shielding [39] inflate the heat of ablation.

On (ii), the distance residual heat spreads before the neighbouring pulse arrives is characterised by the thermal diffusion length:

$${\ell}=2\sqrt{\alpha t}$$

where thermal diffusivity (α) is

$$\alpha =\frac{k}{\rho c}$$

and *t* the interval to the neighbouring pulse. Thermal conductivities of 0.28–0.68 W/mK for human and bovine bone[38, 40, 41], with the density and specific heat above, give a thermal diffusivity ranging from 0.1–0.23 mm^2^/s. The resulting diffusion lengths for the fast and slow axis of each strategy are listed in Table 4.

**Table 4:** Fast-axis and slow-axis thermal diffusion lengths across targeting patterns

Pattern	Fast-axis diffusion length (µm)	Slow-axis diffusion length (µm)
Hatching	30–50	220–340
Interlaced lines	30–50	670–1020
Low-dispersion scatter	180–280	550–840
High-dispersion scatter	510–770	880–1330

These lengths can be compared with the dimensions of the volume the subsequent overlapping pulse ablates: the spot radius laterally (250 µm) and the layer depth axially (300–530 µm), which is achieved from several overlapping pulses. On the fast axis of hatching and interlaced lines, 𝓁 = 30–50 µm is roughly an order of magnitude smaller than both dimensions, so little residual heat escapes the volume the subsequent pulse ablates. In both scatter strategies, the diffusion length is comparable to or greater than the crater dimensions on both axes (180–770 µm fast, 550–1330 µm slow), so a substantial fraction of the residual heat diffuses beyond the ablation volume before the next overlapping pulse arrives.

This is consistent with residual heat contributing to ablation. Hatching and interlaced lines were both more efficient than the scatter strategies, significantly so in every comparison except interlaced lines versus low-dispersion scatter (*p* = 0.061; Table 3). Because the fast axis has the shortest interval between overlapping pulses, it retains the most residual heat that can contribute to ablating a larger volume. Supporting this, hatching and interlaced lines share the same fast axis and differ threefold in slow-axis diffusion length yet showed no significant difference in efficiency (*p* = 0.332).

Where heat escapes the ablation volume, each pulse must re-supply the energy to reach threshold, removing less material and leaving more energy in the bulk tissue. Over the long intervals between pulses, this heat likely spreads through the surrounding bone rather than being ejected, appearing not as a high instantaneous peak but as an elevated mean surface temperature that accumulates over successive passes. Accordingly, the scatter strategies reached the highest mean surface temperatures (Fig. 5b) despite lower peaks (Fig. 4b).

An inter-pulse coupling mechanism may also account for the notably higher removal rates and efficiencies observed in the present study compared with the existing Er:YAG bone ablation literature [14]. Many prior investigations have employed low repetition rate Er:YAG systems which produced consequently lower ablation efficiencies (0.06–0.16mm^3^/J) even with similar pulse duration and fluence [42–44]. The 350 Hz operation used, combined with the hatching strategy reduces the interval between successive pulses at adjacent sites to the order of milliseconds, allowing strong inter-pulse thermal coupling that is not attained in these systems with lower repetition rates.

If this hypothesis is correct, increasing pulse repetition rate should amplify inter-pulse coupling effects, potentially yielding nonlinear improvements in ablation efficiency as the interval between successive pulses at adjacent sites decreases further. This suggests a favourable scaling relationship between laser repetition rate and ablation performance that warrants investigation as higher frequency Er:YAG sources become available.

Several limitations of the current study warrant consideration. All experiments were performed on ex vivo lamb femur specimens thawed to approximately 20 °C, rather than at physiological temperature (37 °C). As a result, absolute decay times and recovery dynamics should be interpreted cautiously, as baseline temperature and convection conditions influence both cooling rate and the temperature–time dose delivered to tissue. Vascularised tissue in vivo would additionally exhibit convective heat dissipation through perfusion, introducing competing thermal transport mechanisms not captured in this model.

The trabecular bone of skeletally immature lambs closely resembles human proximal tibial trabecular bone in density and composition [37, 45], with apparent density, mineral and collagen content nearly identical to donors aged 60–83 years, the population most relevant to arthroplasty. However, we remark significant difference in mechanical strength: relative to this group, lamb bone has roughly twice the ultimate stress and three times the failure energy. Removal rates in human cancellous bone would therefore be expected to be comparable, if not higher. The denser cortical and sclerotic bone encountered in arthritic joints would likely ablate slower, though the relative ranking of targeting strategies is unlikely to change. Characterising performance in these tissues is an area for further investigation.

The mechanisms underlying inter-pulse thermal coupling in thermomechanical ablation warrant further investigation. Targeting strategies that more intentionally explore inter-pulse coupling, such as Hilbert curves and Archimedean spirals, while controlling for tissue thermal properties, would allow deeper insight into the role thermal diffusion plays in ablation. High-speed visualization of the ablation plume dynamics could reveal whether thermally pre-conditioned tissue produces different ejection characteristics, while finite-element thermal modelling could quantify the spatial extent and temporal evolution of inter-pulse thermal fields. These findings could also guide the planning of larger clinical bone preparations, where efficiency must be balanced against the risk of thermal damage. Dividing complex preparations into discrete processing blocks, with pulses in each block ordered to maximise coupling and the blocks sequenced to limit global heat accumulation, is a practical implication of this work that merits further study.

## Conclusion

This study investigated spatial targeting patterns for Er:YAG laser bone ablation and found ablation efficiency to vary with pulse order, indicating a dependence beyond single pulse dynamics. Across strategies delivering the same number of pulses to the same grid locations, efficiency ranged from 0.19 to 0.32mm^3^/J. We propose inter-pulse thermal coupling, whereby residual heat from a preceding pulse remains at the overlapping site of the next and lowers the energy required to reach ablation threshold. Varying pulse separation showed layer depth to scale linearly with deposited energy, so pulse density can be used to set layer depth with no measurable reduction in removal rate across the separations tested. Optimising the targeting strategy achieved the fastest reported removal rate for cancellous bone, 559mm^3^/min in ex vivo ovine bone, identifying targeting strategies as a possible lever for faster ablation.

## Supplementary Information

Supplementary file 1.

## Data Availability

The data supporting the findings of this study are included in the published article. Additional datasets generated or analysed during the study are available from the corresponding author on reasonable request.
